# O7.3. DOSE-RESPONSE META-ANALYSIS TO IDENTIFY THE OPTIMUM AND EQUIVALENT DOSES OF ANTIPSYCHOTIC DRUGS FOR SCHIZOPHRENIA

**DOI:** 10.1093/schbul/sby015.232

**Published:** 2018-04-01

**Authors:** Stefan Leucht, Alessio Crippa, Nicola Orsini, John Davis

**Affiliations:** 1 Technical University of Munich; 2 Karolinska Institutet; 3 University of Illinois at Chicago

## Abstract

**Background:**

It is important to better understand the optimum doses and equivalent doses of antipsychotic drugs. Several methods to understand these relationships have been published, but all these methods have weaknesses. In this paper we present a dose-response meta-analysis which theoretically is the most appropriate method for this purpose.

**Methods:**

We identified all double-blind, placebo-controlled, studies that compared at least two fixed doses of second-generation antipsychotic drugs or haloperidol in people with acute schizophrenia or with predominant negative symptoms. For this purpose, we searched multiple electronic databases, the website of the FDA, and the clinical trial database clinicaltrials.gov. The method applied was dose response meta-analyses with a spline model. The outcome was the reduction of the PANSS or BPRS total score from baseline or – for negative symptoms - a negative symptom scale. With this method we identified 95% effective doses (these have also been called “near-to-maximum” doses). We applied linear splines to examine whether the dose-response curves had already reached a plateau. Moreover, the identified dose-response relationships of each drug were used to derive risperidone equivalent doses.

**Results:**

We identified 67 randomized-controlled trials that were eligible. The following 1mg risperidone equivalent doses were identified: amisulpride 86.6mg/day, aripiprazole 1.9mg/day, asenapine 2.4mg, brexpiprazole 0.56mg clozapine 91mg, haloperidol 1.01mg, iloperidone 3.2mg, lurasidone 23.5mg, olanzapine 2.4mg, paliperidone 13.4/2.1, quetiapine 77mg, risperidone 1mg, sertindole 3.6mg, ziprasidone 30mg.

**Discussion:**

From a conceptual point of view, dose-response meta-analysis is the most appropriate method to identify maximally effective doses and equivalent doses. The results of this meta-analysis will be compared with other published methods to define dose-response, in particular the minimum-effective dose method, the classical mean dose method, the daily-defined-doses (DDD) method and expert consensus methods. The results of this analysis are likely to provide information with impact for treatment decisions.

